# Concordance between dual indirect methods for assessing fat percentage

**DOI:** 10.2478/joeb-2024-0004

**Published:** 2024-04-04

**Authors:** Hurtado B., Colina E., Gonzalez-Correa C. H.

**Affiliations:** 1 Clinical Department, University of Caldas, Manizales, Colombia; 2 Department of Physical Action, University of Caldas, Manizales, Colombia; 3 Basic Sciences for Health Department, University of Caldas, Manizales, Colombia

**Keywords:** body composition, adipose tissue, electrical impedance, anthropometry

## Abstract

In the last decades there is a growing interest in the evaluation of human body composition for being an important part of the integral assessment of individuals. Its use has been extended to different disciplines associated with health care (medicine, nutrition, physiotherapy), and to sports and population fields. Specifically, fat percentage can be related to innumerable diseases. However, there are discrepancies in the results of fat percentage measurement measured by different methods.

**Objective:**

To evaluate the concordance between two low-cost and easily accessible double indirect methods, which have been used indistinctly in different studies where access to more accurate methods is not available, and to determine fat percentage and its relationship with age, sex, body mass index (BMI), waist circumference, level of physical activity and sedentary hours.

**Materials and Method:**

Twenty-four persons between 18 and 38 years and 28 between 39 and 59 years from a university community were evaluated. Calculations were made: BMI, fat % was estimated by anthropometry with a digital adipometer (Skyndex System I USA) and by Electrical Bioimpedance Analysis – BIA (Biody Expert ZM II FRA), physical activity level and sedentary hours were determined with the short IPAQ questionnaire. Pearson's correlation coefficient, Bland & Altman's graphical method and Lin's concordance correlation index were calculated. The significance level p<0.05 was estimated.

**Results:**

The fat percentage by anthropometry was: 30.5% ±8.5 (18–38 years) 35.0% ±6.7 (39–59 years); by BIA 27.0% ±7.3 (18–38 years) and 29.2% ±7.0 (39–59 years). Both techniques showed good correlation, but low degree of concordance (Lin index less than 0.9) except for the group of young persons with moderate level of physical activity (0.95).

**Conclusions:**

The doubly indirect methods used in the study showed strong correlation, but low concordance, so their use is not recommended indistinctly for the follow-up of a particular case. According to the study data for this specific population in young people with moderate physical activity, follow-up could be performed with either of the two methods.

## Introduction

The study of body composition is the assessment of body components (fat mass and fat-free mass) in relation to total body weight and has been considered as a good expression of the effects related to diet, physical activity, diseases suffered during life and finally of changes and adaptations related to the environment. There are multiple methods for the assessment of body composition that vary in access, cost and safety [1]. Currently, worldwide, there are important changes in habits and lifestyles, characterized by insufficient physical activity, diets rich in saturated fats and refined sugars and poor in micronutrients. These elements, combined with the growing supply of information and communication technologies, which favor poor physical activity and an increase in sedentary behavior, have been associated with an increase in chronic noncommunicable diseases (NCDs), which are associated with increased morbidity and mortality and impoverished health quality of the population at all socioeconomic levels throughout the life cycle [2]. The global action plan for the prevention and control of NCDs suggests that intervention on common risk factors such as smoking, unhealthy diet, physical inactivity, and harmful use of alcohol could achieve a 25% reduction in global mortality from cardiovascular disease, diabetes, cancer, and respiratory diseases by 2025 [2]. Prevention is the most valuable and feasible tool for controlling the double burden of malnutrition and physical inactivity, which together impose a high cost on health systems, especially in poor and/or developing countries [2].

Health policies that create enabling environments to ensure access to and availability of healthy choices are essential to motivate people to adopt and maintain healthy behaviors. The low demand for physical activity in modern life and increased sedentary time accelerate the processes of deteriorating physical capabilities and changing body composition (CC). In the Global Burden of Disease Study 2017 [3], globally 61% of deaths and 48.3% of disability-adjusted life years (DALYs) were attributed to factors such as high blood pressure, smoking, and high plasma glucose levels. fasting and high body mass index. Therefore, having a method to assess body composition that is low cost, easy to use, reliable, safe, requires minimal training and can be applied at any level of health care would represent a great advance in the comprehensive evaluation of the individual, particularly if it allows the assessment of the percentage of fat, a component directly related as a risk factor for cardiovascular and metabolic diseases. There are different methods for the evaluation of body composition and especially fat percentage: There are methods considered precise and accurate to measure the different body components, but they have limitations for ambulatory and population use due to costs and/or technological requirements; on the other hand, there are less precise methods, but they have advantages of low cost or few requirements [4].

Different studies have been carried out which show differences in the results of the fat percentage determined by double indirect methods. In the meta-analysis performed by Bohm and Heitmann *et al*., 55 studies were analyzed in healthy people in an age range of 6 to 80 years, concluding that there are important limitations in the comparisons of the results of body composition, especially the fat percentage between bioimpedance analysis and BMI (5). A study conducted by Lizana *et al*., in students aged 10–18 years, evaluating the fat percentage estimated by anthropometric measurements and manual bioimpedance, according to gender and adiposity rate, concluded that the use of manual bioimpedance is not recommended as an interchangeable method with anthropometric measurements in children and adolescents, since bioimpedance underestimates the percentage of fat mass [6].

On the other hand, the study carried out by Ortega Gonzáles *et al.*, in university women, comparing body composition by bioimpedance analysis and anthropometry, found concordance using the Bland & Altman method, when the range of 10% of the upper and lower limit is not exceeded and the predictive formulas are in accordance with the recommendation of the authors; Siri for body fat, Poortamans for muscle mass and Watson for body water [7].

The present study aims to contribute to the discussion on the concordance between two doubly indirect methods, bioimpedance and anthropometry, to determine the fat percentage, using the Bland & Altman graphs and the Lin index as statistical tools, and to establish the relationships with different variables such as sex, age, BMI, level of physical activity and sedentary hours.

## Materials and methods

This was an observational, non-experimental, cross-sectional, relational study.

### Population and sample

A sample of 52 people belonging to the University of Caldas was collected (students, teachers and employees). They were between 18 and 59 years, 35 women (67.3%) and 17 men (32.7%). Exclusion criteria were the following conditions: edema, pregnancy status, menstrual period, presence of amputations, regular use of diuretics or glucocorticoids, paralysis of the extremities of any origin, presence of implanted electronic equipment (pacemakers or resynchronizers), presence of medical or cosmetic prosthesis or active skin lesions or wounds.

Measurements were taken by a single investigator and under the same conditions (in underwear, without footwear and devoid of metal objects) for anthropometric measurements; BMI was calculated and categorized according to the WHO level (underweight, normal weight, overweight, obesity I, obesity II, and obesity III). Subcutaneous folds were taken on the right side, following the protocols of the International Society for the Advancement of Kinanthropometry (ISAK) for marking and determining the site of fold measurement. The calculation of the fat percentage was performed automatically with the Skyndex System I equipment (USA) calibrated according to age and sex, using the Durnin and Womersley formula for the general population.

For the electrical bioimpedance analysis, the Biody Expert ZM II (FRA) equipment was used, following the protocol of Gonzales-Correa *et al.* [8] and according to the manufacturer's recommendations [9–11] and the level of fat percentage was categorized, according to the studies of Gallagher *et al*., into low, normal, high and very high [12]. The level of physical activity (low, moderate and vigorous) and the hours sedentary/week were determined by applying the International Physical Activity Questionnaire - IPAQ short, validated in Spanish [13].

For the statistical analysis, the normality of the variables was established using the Shapiro-Wilk test. The relationship between the percentage of fat measured by bioimpedance and anthropometry with the other variables was determined by Pearson's correlation coefficient (for normal variables) or Spearman's correlation coefficient (otherwise). Dependence between qualitative variables was established by Pearson's chi-squared test. For the analysis of concordance, Lin's concordance correlation coefficient was used, which combines the measure of precision and the measure of accuracy, and Bland & Altman's graphic analysis, which makes it possible to evaluate the bias between mean differences and to estimate an interval of agreement within which 95% of the differences of the second method with respect to the first are located [14]. For all analyses the significance level p <0.05 was used.

### Informed consent

At the time of sample collection, the route established in the institution’s Biosafety Protocol COVID-19 was followed and the informed consent form was filled out.

### Ethical approval

From the ethical point of view, the study complied with the international regulations on biomedical research of the Declaration of Helsinki [15], in addition, it followed the national regulations according to Resolution 8430 of 1993 and was approved by the Ethics Committee of the University of Caldas, being considered research with minimal risk.

## Results

Fifty-two people were evaluated, 35 women (67.3%) and 17 men (32.7%) with an average age of 37.6 ±11.4 years. By the trends of the results, they were divided into four groups, women 18–38 years (51.4%) older than 38 years (48.6%) and men younger than 18–38 years (35.2%) and older than 38 years (64.8%).

Table 1 shows the anthropometric variables. The differences in BMI for 18-38 years (24.3 + 3.3) and for those over 39 years (27.2 + 3.7) were significant. Regarding the level of physical activity, 50% of the younger subjects reported low levels of physical activity, while in the older subjects this figure rose to 64.3%; however, the differences were not statistically significant. Likewise, no significant differences were found in the number of sedentary hours between the two age groups (6.8 hours for young people and 8.4 hours for adults).

**Table 1.** Quantitative variables by sex-age

Sex	Age	Nº	BMI	Fat % Ant	Fat % Bio	P value comparing A/B
**Figure:** 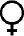	18 to 38	18	24.6 (3.5)	34.4 (5.1)	30.1 (5.1)	**<0,0001**
	> 38	17	27.1 (4.1)	38.6 (5.1)	33.4 (5.5)	**<0,0001**
P value by age females			0.056	**0.021**	0.070	
**Figure:** 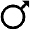	18 to 38	6	23.4 (2.5)	19.1 (5.5)	17.8 (4.5)	0.225
	> 38	11	27.5 (3.2)	29.5 (5.0)	23.0 (3.4)	**0.002**
P value by age males			**0.018**	**0.001**	**0.016**	

The level of fat percentage measured by both methods correlated in women of both age groups and men >38 years (0.0001 and 0.002) but not in men aged 18 to 38 years All groups correlated with BMI.

To determine concordance, Lin's concordance correlation coefficient was calculated between the fat percentage measured by anthropometry and by bioimpedance, discriminating by sex, age, level of physical activity and BMI (Table 2). According to the Lin concordance coefficients found, all showed poor concordance (less than 0.9), with the exception of the one reported in young people who report moderate physical activity (0.9463). It is also striking that these coefficients are higher for all cases in younger people.

**Table 2.** Lin's coefficient of concordance

Variable	Categories	#	Lin's coefficient	Lower limit (95%)	Upper limit (95%)
Age	<	24	0.86	0.73	0.92
	>	28	0.60	0.38	0.75
Sex	♀<	18	0.66	0.41	0.81
	♀>	17	0.51	0.22	0.72
	♂<	6	0.86	0.39	0.97
	♂>	11	0.12	0.00	0.38
Physical activity level	Sedentary <	12	0.81	0.56	0.93
	Moderate <	3	0.95	0.64	0.99
	High <	9	0.81	0.54	0.93
	Sedentary >	18	0.64	0.39	0.81
	Moderate >	5	0.82	0.14	0.97
	High >	5	0.00	0.00	0.30
BMI	Normal weight <	13	0.82	0.62	0.92
	Overweight <	10	0.72	0.42	0.88
	Obesity I <	1	-	-	-
	Normal weight >	9	0.38	0.00	0.69
	Overweight >	12	0.53	0.16	0.77
	Obesity I >	7	0.73	0.29	0.92
Waist circumference	Normal <	13	0.84	0.65	0.93
	Abnormal <	11	0.71	0.37	0.88
	Normal >	7	0.43	0.00	0.79
	Abnormal >	21	0.53	0.28	0.72

**Figure 1.**
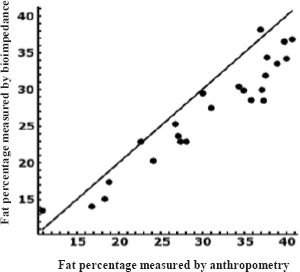
Graph of concordance between % fat measured by anthropometry and % fat measured by bioimpedance in people from 18 to 38 years old. Lin's index. (0.855).

**Figure 2.**
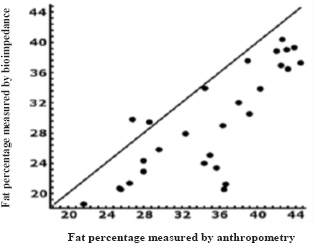
Concordance between % fat measured by anthropometry and % fat measured by bioimpedance in people older than 38 years. Lin's index. (0.597).

Figures 1 and 2 show the concordance between the two methods, observing a dispersed distribution of the data with a tendency to concentrate below the line of perfect concordance, so it is interpreted that the measurements made by anthropometry are higher than those made by bioimpedance in both, differentiated by age groups.

**Figure 3.**
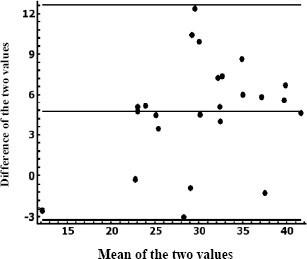
Bland & Altman graph for agreement between % fat measured by anthropometry and bioimpedance for people aged 18–38 years.

Figure 3 shows the Bland & Altman graph for people under 38 years of age, where it is observed that all the data are between the limits of agreement, which indicates a stability index of 100%. This suggests that there is some agreement between both methods for estimating body fat in these individuals. However, the limits of agreement are broad.

**Figure 4.**
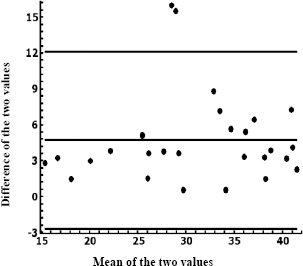
Bland & Altman plot for agreement between % fat measured by anthropometry and by bioimpedance over 38 years old.

Figure 4 shows two points outside the limits established by the Bland & Altman method for the percentage of fat measured in people over 38 years of age, which allows us to establish a stability index of 92.9%.

It is observed in the graph that the distribution of the data was uniform and that the mean is about 4.7 ±3.96, with an interval of –3.2 to 12.6 of variation of the estimation of the percentage of fat, (which in the authors' opinion are too wide limits for a variable such as the percentage of fat). In other words, anthropometry overestimates the measurement (fat percentage) with respect to electrical bioimpedance.

## Discussion

Considering that the percentage of fat is strongly related to increased cardiometabolic risk of different types of cancer (colon, endometrium) and musculoskeletal diseases [16,17], it becomes a negative factor in public health.

In this context, it is important to be able to count on a tool that facilitates the evaluation of body composition and the determination of the fat percentage, allowing clinical evaluation at any level of health care. Of the available tools, the most easily accessible are anthropometry and electrical bioimpedance, which have not shown inferiority to direct methods such as DXA and air displacement plethysmography [18], for this reason multiple studies have been conducted to determine the concordance between these two methods.

In the present study it was observed that despite the differences between the two methods, there is a good correlation between them. These findings are in agreement with what was reported in studies conducted on university women [7] and the one conducted in 2018 in adult and older adult women [19]. However, when determining the agreement between methods there is a great discrepancy in the literature, with studies that report agreement between methods, such as those performed by Ortega-González where the analysis of different formulas for the calculation of the fat percentage used from skinfolds and electrical bioimpedance was performed, finding a good agreement between the Siri equation and bioimpedance [7]. In another study, performed in women aged 19–67 years, concordance is also reported, using the graphic method of Bland and Altman [18], between the fat percentage calculated by anthropometry using seven subcutaneous folds, and that determined by bioimpedance using seven different set-ups and comparing them with the gold standard, air displacement plethysmography (ADP).

Finally, the study conducted by Silveira with adults over 60 years of age (in whom changes in body composition associated with the aging process are expected), comparing the two methods versus DXA, found that in all participants there was a strong agreement between the fat percentage calculated by skinfolds, using the Durnin and Womersley equation and bioimpedance, the statistics used was Lin's correlation coefficient concordance. However, reviewing the data provided by the authors and according to the interpretation of the coefficient it would give a poor level of concordance (0.857 and 0.861) [14]. In this study they found that both methods underestimate the fat percentage in both men and women with high body fat percentage compared to the reference method (DXA) [20].

A study conducted on physically active young subjects of both sexes found low concordance in men and good concordance in women, using the intraclass correlation coefficient and the Bland and Altman graphical method. For anthropometric analysis, they used the Whiters formula for density and the Siri formula for fat percentage, and four different bioimpedance devices. The devices that showed the highest concordance were Inbody 720 and Tanita BC400. [21].

On the other hand, the study carried out by Moreno *et al.* in a population of healthy adults determined the percentage of fat by anthropometry using different formulas and the analysis of electrical bioimpedance, concluded that the calculation of the percentage of fat by anthropometry is not concordant with the data obtained by bioimpedance, using the Bland and Altman graph, and concordance was only found between bioimpedance and the Siri-specific equation [22].

A study carried out in Cuban older adults to evaluate the concordance between anthropometry, electrical bioimpedance and dilution with deuterium, concluded that bioimpedance tends to overestimate the fat percentage with respect to deuterium, while anthropometry tends to underestimate the fat percentage with respect to the reference standard. Among the doubly indirect methods, they found that bioimpedance overestimated the amount of total fat. With respect to our study, no concordance was found between the methods. On the contrary, anthropometry overestimated the fat percentage with respect to bioimpedance, clarifying that in the Cuban study a monofrequency bioimpedance equipment was used [23] and it has been documented that multifrequency equipment is more precise to differentiate the variations in the hydration state [24]. The study conducted by Hernández-Ruiz in middle-aged women found low concordance, measured with the Kappa index (0.397), comparing the fat percentage content measured by triceps skinfold, bipolar bioimpedance and DXA [25].

In the Colombian population, a study was carried out with healthy adults of both sexes, determining the fat percentage by measurement of four folds using the Durnin and Womersley and Jackson/Pollock equation compared with the foot–foot bioimpedance measurements using the Kappa index, concluding that there are significant differences between methods and between anthropometric equations, suggesting that the results are not comparable or interchangeable [26]. The differences found in the measurements between methods can lead to a misinterpretation of the classification of fat percentage level.

In the studies found in the literature, it is observed that the statistical analyses use tools to evaluate correlation and concordance indistinctly, which makes comparison between studies difficult and may be a reason for the differences. Furthermore, the studies were carried out in very specific populations (by age, sex or physical activity), for example, the studies where concordance between methods is found were generally designed in a young population with regular practice of physical activity, which does not allow extrapolating the results to the general population, and concordance between methods has not been found in patients with overweight and obesity [27].

A significant correlation was found between the two methods, according to the analysis of the Bland & Altman stability index, frequently used in medical literature [28]. Even so, the dispersion of the points is wide, and it is difficult to establish an acceptable dispersion range. This is where clinical judgment and analysis of the results of this estimation come into play [28].

On the other hand, using Lin's concordance correlation index, currently considered more rigorous from the statistical point of view, an acceptable correlation was only demonstrated in young people with a moderate level of physical activity (0.95). In the reviewed literature, neither the physical activity variable nor sedentary behavior measured as sedentary hours was found as a factor in favor or against the concordance between the methods, so it cannot be compared with other studies.

Although the present study was carried out on a small sample and for convenience, the results constitute a contribution to research on the relevance of defining the concordance between two methods that are easy to use, low cost and more accessible in developing countries with limited health resources. Finally, it should be noted that it was not in the interest of the researchers to determine the validity of the methods in the absence of a reference method.

## Conclusions

The two methods showed strong correlation, but low concordance and can only be interchangeable, for this sample, in young people with moderate physical activity.

It can also be concluded that the two double indirect methods for determining the fat percentage may behave differently in other types of populations and that, given the results, in clinical practice it is recommended to become familiar with only one method, whichever of the two is available, and to always perform clinical follow-up with the same method.

Finally, attention should be paid to the statistics used in concordance studies to improve interpretation and allow comparison of the results.
